# Induction Strategy for Locally Advanced Thymoma

**DOI:** 10.3389/fonc.2021.704220

**Published:** 2021-07-22

**Authors:** Yang Zhang, Zongjuan Li, Yixing Chen, Lijie Tan, Zhaochong Zeng, Jianyong Ding, Shisuo Du

**Affiliations:** ^1^ Department of Radiation Oncology, Zhongshan Hospital, Fudan University, Shanghai, China; ^2^ Department of Thoracic Surgery, Zhongshan Hospital, Fudan University, Shanghai, China

**Keywords:** locally advanced, thymoma, induction therapy, neoadjuvant, R0 resection

## Abstract

Surgery remains cornerstone for the management of thymoma. Complete surgical resection (R0), is recognized as the constant and significant factor for prognosis. However, in locally advanced (Masaoka-Koga stages III-IVa) thymomas, achieving R0 resection remains challenging due to local-regional invasion of the disease. Induction treatment, with the aim of reducing bulky tumor mass, offers new strategy to facilitate totally surgical resection. Herein, we reviewed recent progress and provided a comprehensive overview of induction strategy in locally advance thymoma.

## Introduction

Thymic epithelial neoplasms, commonly comprising thymoma and thymic carcinoma, are rare tumors with a broad spectrum of biological behavior. Unlike thymic carcinomas, thymomas are relatively indolent. Once they are completely resected (R0), long-term survival may be easily achieved (1–3). Thereby, only thymomas were considered for this review.

According to published data, approximately 30% thymomas are staged as Masaoka-Koga III-IVa, and for these patients, performing R0 resection is not easy, due to locoregional extent of the disease (4, 5). Given the prognostic benefits of R0 resection, induction strategies, including chemotherapy, radiotherapy and even targeted drugs, have been explored, and some reviews have also been published during last decade (6, 7). However, in recent years, chemo regimens and radiotherapy technologies are rapidly evolving, immunotherapy and targeted drugs are fast-growing. Knowledge regarding induction strategy in thymoma, requires updating. So, we conducted this narrative review, discuss the current progress and future directions in the setting of induction treatment of locally advanced thymoma.

## Induction Chemotherapy: Theoretical Advantages and Clinical Practices

Induction chemotherapy is a priority criteria for initially unresectable thymomas. Firstly, this is because thymoma is revealed as chemo-sensitive (8, 9); Secondly, chemotherapy is easily to perform and with limited toxicity. Theoretically, induction chemotherapy presents several advantages. Foremost, induction chemotherapy can result in tumor shrinkage, which then improves the probability of R0 resection. Further, the systematic micro-metastasis of the neoplasm may be inhibited at an early stage. Patients show a better tolerance to induction therapy compared with adjuvant chemotherapy. Ultimately, the objective response to induction treatment may provide information to help guide post-operative chemotherapy (10–12). However, to transform theory into reality, the following questions must be answered.

### What Is the Exact Role of Induction Chemotherapy in Clinical Practice?

The exact role of induction chemotherapy in locally advanced thymoma patients still remains controversial. Given the rarity of the tumor, there have been no randomized controlled trials conducted to date. Nonetheless, some studies may provide insight into the effects of induction chemotherapy treatment.

Yamada et al. (13) using a Japanese Nationwide Database retrospectively explored the role of induction chemotherapy in stage III thymoma, surprisingly the authors determined that induction chemotherapy was an adverse prognostic factor. The authors proposed that induction treatment in their study was correlated to larger radiological tumor size, a higher number of involved sites, and invasion into the phrenic nerve. Thus, induction therapy itself did not worsen prognosis but acted as a selection bias. In addition, the small sample size may have represented another reason for this phenomenon.

Most recently, Khorfan et al. (3) retrospectively analyzed the management of advanced thymoma in the United States, a total of 160 patients (128 patients received induction chemotherapy) were administered induction treatment. As in the study by Yamada et al., patients with more advanced unresectable disease were more likely to be chosen for induction treatment. However, R0 resection rates in the induction group, were similar to those of patients receiving surgical intervention (57.2% *vs.* 54.2%), which may indicate the role of induction chemotherapy in facilitating R0 resection.

To eliminate the influence of confounding biases, a propensity score-matched analysis was performed. Thymoma patients receiving induction chemotherapy followed by surgery and patients receiving surgery alone had similar resectability, overall survival, and recurrence-free survival rates (14). In addition, Cardillo et al. (12) conducted a study to evaluate the prognostic factors in locally advanced thymoma and thymic carcinoma. In their study, 30 patients received immediate surgery, 31 patients ineligible for surgery at presentation underwent induction chemotherapy followed by surgery. Patient characteristics in these two groups were well-balanced. The results showed that the 10-year survival rates in the induction group were better than those for the immediate surgery group (57.9% *vs.* 38.1%, p=0.03).

Bretti et al. (15) performed a study in Masaoka-Koga stages III-IVa malignant thymoma patients. Thirty patients received surgical treatment, while 25 cases, ineligible for radical surgery, received induction chemotherapy before surgical reassessment. Following induction chemotherapy, 14 patients (56%) underwent surgery, and 11 cases (78.6%) achieved a complete resection. The study revealed that the overall survival of individuals who initially performed R0 resection was better than those failing to achieve R0 resection. Furthermore, the survival outcomes in the R0 resection subgroup after induction chemotherapy approximated that of direct R0 resection patients.

Despite the paucity of clinical data confirming the importance of induction chemotherapy, the existing evidence strongly suggests that induction chemotherapy is a preferred choice for initially unresectable thymoma patients.

### Which Scheme Is Commonly Recommended for Induction Chemotherapy?

Currently, the optimal chemotherapy regimen remains controversial. Apart from induction therapy, patient prognosis can be influenced by surgical intervention, the adjuvant treatment strategy, and several other confounding factors. Thus, this review will place a greater focus on treatment response and the R0 resection rate to compare different chemotherapeutic regimens.

#### Cisplatin and Etoposide-Based Treatment Regimens

Hassan et al. (16) performed a study including 9 patients with unresectable (III and IVa) thymic tumors. Patients underwent induction chemotherapy with 3 courses of cisplatin and etoposide (EP). One (11%) patient achieved a complete remission (CR), 6 (66%) a partial remission (PR), and the remaining 2 (22%) patients, achieved only a minor remission. After induction therapy, 1 patient refused surgery and 5 (62.5%) of the other 8 achieved a R0 resection.

Macchiarini et al. (9) incorporated epirubicin into a cisplatin and etoposide regimen (PAE). Nine clinically staged IIIa invasive thymoma patients were enrolled in the analysis. After 3 cycles of treatment, all patients (100%) achieved a PR and 4 of the 7 (57.1%) patients were subjected to R0 resection. Lucchi et al. (17) also explored the role of PAE regimen as an induction scheme, in his study, treatment response and R0 resection rates were 73.3% and 76.7% respectively.

#### Platinum and Anthracycline-Based Treatment Regimens

The platinum and anthracycline-based scheme is most widely prescribed in clinical practice, and the following combinations were included: cisplatin+doxorubicin+cyclophosphamide (PAC), cisplatin+doxorubicin+cyclophosphamide+prednisone (PACE), cisplatin+adriamycin +cyclophosphamide+vincristine (ADOC), and cisplatin+doxorubicin+methylprednisolone (CAMP).

Shin et al. (18) conducted a prospective study which included 12 unresectable III-IVa thymoma patients, in which all of them received 3 courses of PACE. Three patients (25%) achieved a complete tumor response and a partial response was obtained in 8 patients (67%). One patient refused the subsequent surgical intervention, while for the remaining 11 patients, R0 resection was achieved in 9 (81.8%) patients. Kim et al. (19) also explored the role of PACE in III-IVb invasive thymoma. All patients included in the study received 3 courses of chemotherapy, and major responses were observed in 17 (77%) of the 22 patients, including 3 (14%) CRs and 14 (63%) PRs. After induction treatment, 1 patient refused surgery, but the remaining 21 patients were treated surgically, and of these, 16 (76%) achieved a R0 resection.

Sixteen clinically staged III-IVa invasive thymoma were enrolled in a study from January 1985 to November 1991, all patients were treated with the ADOC scheme. The response rates for ADOC induction therapy were 100% (7 CRs and 9 PRs). Subsequently, all the patients underwent surgery and 11 patients (68.8%) achieved a complete resection (20). Another clinical trial including 16 consecutive patients with stage III and IVa invasive thymoma, also evaluated the effects of ADOC induction chemotherapy, 13 (81.3%) patients responded well to the regimen. All 13 patients responding well were directed to surgery, and for 9 (69.2%) patients, tumors were radically resected (21).

CAMP is also widely used in clinical trials. In a study conducted by Kohei et al. (22), the CAMP regimen was administered in the neoadjuvant setting in 14 invasive III-IVb stage thymoma patients. All patients except 1 (92.9%) showed a good response to this scheme. Finally, surgical treatment was performed in 9 patients and 2 (22.2%) achieved a R0 resection.

#### Other Combinations

Park et al. (23) explored the role of induction chemotherapy using docetaxel and cisplatin (TP). In their study, 9 (1 stage III and 8 stage IVa stage) thymoma patients were enrolled. After receiving induction chemotherapy, 5 (55.6%) patients achieve a PR and 4 patients responded stably to the scheme. Following reassessment, 7 patients became eligible for surgery and all (100%) ultimately achieved a R0 resection. Detailed information about induction chemotherapy regimens is listed in detail in **Table 1**.

**Table 1:** Induction chemotherapy for locally advanced thymoma.

Author	Sample size	Stage	Pathology	Chemo regimen	Response rate % (CR+PR)	No. of R0/No. of surgery (%)
Macchiarini et al. (9)	7	IIIa	thymic tumor^*^	PAE	100%	4/7 (57.1%)
Lucchi et al. (17)	30	III-IVa	thymoma	PAE	73.3%	23/30 (76.7%)
Hassan and Seoud (16)	9	III-IVa	thymoma	EP	77%	5/8 (62.5%)
Kunitoh et al. (24)	21	III	thymoma	PAE+vincristine	62%	9/13 (69.2%)
Berruti et al. (8)	6	III-IVa	thymoma	ADOC	83.3%	-/5 (-%)
Rea et al. (20)	16	III-IVa	thymoma	ADOC	100%	11/16 (68.8%)
Berruti et al. (21)	16	III-IVa	thymoma	ADOC	81.3%	9/13 (69.2%)
Tan et al. (25)	14	III-IVa	thymoma	ADOC	100%	9/14 (64.3%)
Bretti et al. (15)	25	III-IVa	thymic tumor^*^	ADOC、EP	72%	11/14 (78.6%)
Leuzzi et al. (26)	11	III-IV	thymic tumor^*^	PAC	81.8%	9/11 (81.8%)
Shin et al. (18)	12	III-IVa	thymoma	PACE	91.7%	9/11 (81.8%)
Kim et al. (19)	22	III-IV	thymoma	PACE	77%	16/21 (76.2%)
Cardillo et al. (12)	41	III-IVa	thymic tumor^*^	PACE	75.6% at least a minimal response	–
Yokoi et al. (22)	14	III-IV	thymoma	CAMP	92.9%	2/9 (22.2%)
Ishikawa et al. (27)	11	IVa-IVb	thymoma	CAMP	75%	4/7 (57.1%)
Nakamura et al. (11)	19	IV	thymoma	CAMP	78.9%	–
Park et al. (23)	9	III-IV	thymoma	TP	55.6%	7/7 (100%)

*including thymoma and thymic carcinoma.

EP, cisplatin+etoposide; PAE, cisplatin+etoposide+ epirubicin; ADOC, cisplatin +adriamycin +cyclophosphamide +vincristine; PAC, cisplatin +doxorubicin +cyclophosphamide; PACE, cisplatin +doxorubicin +cyclophosphamide +prednisone; CAMP, cisplatin +doxorubicin +methylprednisolone; TP, docetaxol+cisplatin; CR, complete remission; PR, partial remission; R0, complete resection.

## Incorporating Radiation Into Induction Chemotherapy

Induction radiotherapy alone is rarely used as initial therapy in thymoma patients (**Table 2**). After screening of National Cancer Database, the largest cancer registry in the whole world, a total of 160 advanced stage thymoma patients received neoadjuvant therapy, however, only 5.7% underwent induction radiation alone (3). According to Chinese Alliance for Research in Thymomas Database, among induction therapy patients, only 13.2% received radiotherapy (31).

**Table 2:** Induction radiotherapy for locally advanced thymoma.

Author	Sample size	Stage	Pathology	RT dose	Response rate % (CR+PR)	No. of R0/No. of surgery (%)
Bretti et al. (15)	8	III-IVa	malignant thymoma*	30 Gy	37.5%	1/3 (33.3%)
Akaogi et al. (28)	12	III-IVb	thymoma	12-21 Gy	91.7%	9/12 (75%)
Yagi et al. (29)	11	III-IV	thymoma	20-66 Gy	–	–
Ohara et al. (30)	6	III-IV	thymoma	12-20 Gy	83.3%	3/6 (50%)

*Detailed information was not shown.

RT, radiotherapy; CR, complete remission; PR, partial remission.

Currently, chemotherapy remains the mainstream induction strategy, meanwhile, in some cases, chemotherapy alone is not sufficiently effective, especially in heavily invasive diseases. Under such circumstances, the potential synergism of combining chemotherapy with radiotherapy, may further potentiate induction response rates and facilitate R0 resection (**Table 3**). Similar to induction chemotherapy, deep consideration should also be given to the following questions.

**Table 3:** Induction chemoradiotherapy for locally advanced thymoma.

Author	Sample size	Stage	Pathology	Chemo regimen	RT dose	Response rate % (CR+PR)	No. of R0/No. of surgery (%)
Wright et al. (32)	10	III-IVa	thymic tumor^*^	EP	40-45Gy	40%	8/10 (80%)
Korst et al. (33)	21	–	thymic tumor^*^	EP	40-45Gy	47.6%	17/21 (77%)
Wang et al. (34)	33	III	thymic tumor^*^	TP	40Gy	78.8%	85.7%

*including thymoma and thymic carcinoma.

RT, radiotherapy; EP, cisplatin+etoposide; TP, docetaxol+cisplatin; CR, complete remission; PR, partial remission.

### What Is the Exact Role of Induction Chemoradiotherapy?

A study conducted by Chu et al., compared radiological response of chemotherapy alone with the combination of chemotherapy and radiotherapy in the management of locally advanced or advanced thymic epithelial tumors (including thymoma and other thymic tumors). An increased average radiological response was observed in the combination treatment group compared with chemotherapy alone (volume: reduction of 47.0 cm^3^ or more, P < 0.001; diameter: a reduction of 0.8 cm or more, P = 0.03), and for patients receiving chemotherapy, further tumor shrinkage was observed in 33% patients when additional radiotherapy or chemoradiotherapy was administered (median volume: 42.3% reduction, P = 0.03; diameter: 3.0% reduction, P = 0.049 (35).

Another study performed by Wright et al., explored the function of induction concurrent chemoradiotherapy in 10 initially unresectable locally advanced thymic tumors (including 7 stage B3 thymomas and 1 thymic carcinoma). The treatment scheme consisted of 2 cycles of EP combined with concurrent radiotherapy (33 to 49 Gy) before surgery. Adjuvant chemotherapy (EP) was administered to incomplete resection patients and for those with high risk of recurrence. After completion of induction treatment, 4 (40%) patients achieved a PR while the remaining 6 patients presented no changes. After reassessment, all 10 patients were directed towards surgery, and an impressive R0 resection was achieved in 8 (80%) patients. After examination of the resected specimens, substantial (>90%) necrosis was observed in 4 (40%) patients. No postoperative deaths were observed and the 5-year estimated survival was 69% (32).

In view of the encouraging results achieved following induction chemoradiotherapy, especially the high pathological response rate, a phase II multi-center study was prospectively conducted. A total of 21 thymic tumor patients (13 thymomas, 7 thymic carcinomas and 1 metaplastic tumor) who met the specific computed tomograph criteria were enrolled. The induction protocol consisted of 2 cycles of the EP scheme and concurrent radiation 40-45 Gy. Ten (47.6%) patients achieved a PR on radiographic assessment, while the remaining 11 showed no response. All patients received surgical treatment, and 17 (77%) of them underwent R0 resection (33).

A study conducted in China examined the induction role of concurrent chemoradiotherapy with a different chemotherapy scheme. In total, 33 patients (10 thymomas, 21 thymic carcinomas and 2 thymic carcinoids) were included. All patients received docetaxel and cisplatin concurrently accompanied by 40 Gy radiation treatment before surgery. After reassessment, the response rate was 78.8% (1 CR, 25 PR, and 7 stable disease [SD] cases). Among the 10 thymoma patients, an 80% response rate was achieved. Finally, 21 patients were recognized as responsive to the surgical procedure, and 18 (85.7%) achieved R0 resection (34).

Due to the differences in the inclusion criteria, the definition of an “unresectable tumor”, and chemotherapy scheme, treatment outcomes in different clinical trials cannot be fully compared. However, a roughly higher rate of R0 resection can still be observed in the induction chemoradiotherapy groups. It may be not appropriate for all thymoma patients to receive induction chemoradiotherapy. Some patients may achieve favorable responses with chemotherapy alone, and furthermore, increased treatment toxicity and the potential risks induced by radiotherapy should not be ignored. Thus, eligible patients should be carefully selected.

### Who Will Benefit Most From Induction Chemoradiotherapy?

#### Patients Presenting Invasion of Great Vessels

Yamada et al. extracted and analyzed data relative to a total of 310 stage III thymoma patients from the Japanese National Database. Among these 310 patients, 126 had great vessel invasion. These cases had significantly lower R0 resection rates compared with patients with no vessel invasion (73.8% *vs.* 83.7%, p= 0.011) (13). Hassan et al. (16) conducted a study to explore the function of induction chemotherapy in locally advanced thymoma patients, 3 cycles of EP were administered before surgery. Of these, only 3 patients presented great vessel invasion before induction chemotherapy, and unfortunately an extensive full-thickness tumor invasion of the vessels was still evident at the time of surgical intervention. Ultimately, all 3 patients achieved an incomplete resection. Thereby, for thymoma with great vessel invasion, chemotherapy alone may be not sufficient, while combining chemotherapy with radiotherapy may enhance the antitumor activity and the possibility of totally resection.

#### Patients With More Invasive Histological Subtype

Onuki et al. (36) performed a study to assess the pathological radioresponse to preoperatively irradiated thymoma. The authors found that type B1 or B2 group had higher reduction ratios than the type B3 group (mean value of 39.7%, 31.8%, and 21.0%, respectively, P < 0.01). One explanation for this phenomenon was that type B1 or B2 presents a larger percentage of radiosensitive lymphocytes compared with B3 tumors. Furthermore, high response rates can also be achieved in type A-B2 thymoma patients who have received either chemotherapy or corticosteroid treatment alone (37). Furthermore, in the study conducted by Korst et al., 21 thymic tumor patients were enrolled to assess the treatment response of induction chemoradiotherapy, and 5 patients ultimately showed near complete pathologic response, and intriguingly, 80% of them were thymic carcinomas (33). Thereby, we may ponder whether preoperative chemotherapy is sufficient for type A-B2 tumors, and for more invasive histological subtypes like B3 thymoma or even thymic carcinoma, induction chemoradiotherapy may represent a better choice.

#### Patients Refractory to the First-Line Induction Chemotherapy

In a study performed by Robert et al., 8 thymic epithelial tumor patients received additional radiotherapy or chemoradiotherapy after the initial chemotherapy. Subsequent radiotherapy further decreased the median tumor volume by 39.9 cm^3^ (P = 0.03) and median tumor diameter by 1.0 cm (P = 0.049) compared to post-chemotherapy measurements (35). Thus, in clinical practice, thymoma patients who are still not amenable to surgery after the first-line induction chemotherapy, additional induction radiotherapy may be a good choice, as it may lead to further tumor shrinkage and facilitate R0 resection. Our institute is performing a prospective clinical trial to further confirm this therapeutic schedule.

### What’s the Role of Radiation Technology Advances in Thymoma?

A major concern about radiotherapy is the undesired dose deposition to the surrounding tissues. In thymoma radiation, this concern is even more pronounced, because surrounding organs, including heart, lungs and esophagus, can not avoid being irradiated. Thymomas patients commonly have long-term survival, and the tumor mass of thymomas requiring induction radiation, is relatively bulky. The above characteristics, provide a strong rational for conducting advanced radiation technology to reduce toxicity. Radiation therapy has made significant technological strides over the past decades. More and more innovative techniques have been applied to the treatment of thymoma, like adaptive radiation therapy (ART), tomotherapy and particle radiotherapy (proton radiotherapy and carbon ion radiotherapy). By the implication of cutting-edge radiation technology, the final goal is to provide a culmination of innovation advances to maximize the therapeutic ratio, and improve R0 resection rate, while minimizing radiation-related toxicity.

The application of ART, or modifying the physical plan during the radiotherapy process, is becoming increasingly available in clinical practice. ART provides powerful potential for minimizing radiation-related injury while escalating or de-escalating target doses based on the dose to organs at risk (38). ART is worthwhile especially in tumors that shrink rapidly during radiation therapy. As reported in the literature, tumor mass of thymoma could be reduced by 40%-78% within the first two weeks of radiation (28, 39). So, ART may have potential role in thymoma treatment. In a pre-clinical study, the dosimetric benefit of ART in neoadjuvant setting of canine and feline thymoma was explored. The research demonstrated that rapid tumor-shrinkage was observed within 1 week of radiation, with a mean shrinkage of 31.0% ± 15.2%, which surly will exert huge adverse impact on normal tissues around the target. After mid-therapy replanning, the dose to organs at risk was significantly reduced, with −18.2% in the mean heart dose and −27.9% in the V20 lung dose (40). Although being promising, the usage of this technology faces the dilemma of lacking enough patients. After screen of papers, only a case report was found. The case showed that induction chemoradiotherapy with ART appears to be powerful weapons for locally advanced intact thymoma (41).

Tomotherapy is the delivery of intensity modulated radiation therapy using rotational delivery of a fan beam in the manner of a computed tomograph scanner (42). To the best of our knowledge, no clinical trial using tomotherapy has been conducted in the field of thymoma. Based on the experiences of our center, tomotherapy will be considered under the following two conditions: 1) complex-shaped thymoma: 3-dimentional radiation therapy or intensity modulated radiation therapy can not achieve satisfactory target volume coverage, or at the sacrifice of organs at risk; 2) multiple tumor lesions: treatment protocol can not be accomplished with single radiotherapy plan. In our institution, tomo is widely used in Masaoka-Koga IVa patients with pleural dissemination. In addition to radiotherapy to the primary site which is not eligible for surgery at initial evaluation, radiation is also delivered to the pleural area. For localized pleural disease, local radiation will be conducted. However, for patients with relative extensive pleural metastases, besides local treatment, prophylactic radiation of ipsilateral entire pleural will also be considered. As we know, most of the recurrence of thymoma occur on the pleural surface (43, 44). So, the aim of the above treatment strategy possesses two purposes: Improve R0 resection rate and reduce the risk of pleural dissemination during operation.

Particle radiotherapy possesses the theoretical dosimetric advantage over photon techniques by the production of “Bragg Peak”, which provides a sharp increase in dose at a given depth in tissue that can be modulated by the treating physician (45). Several studies of lung cancer and Hodgkin lymphoma, have well demonstrated that particle radiotherapy could maintain target dose coverage while minimizing the dose of organs at risk (46, 47). In the field of thymoma, Haefner et al. conducted dosimetric comparison between photon and particle radiotherapy in the postoperative management of thymoma. The results revealed that particle radiotherapy showed superior organs at risk sparing and optimal target volume coverage (48). However, up to now, no data were reported about the implementation of particle radiation in induction radiation of thymoma.

## Adding Immunotherapy to Induction Treatment

Immunotherapy is currently a revolution, as is thymoma. The most commonly used predictor for Programmed Cell Death-1/Programmed Cell Death- Ligand 1 (PD-1/PD-L1) immune therapy is the expression of PD-L1 (49, 50). As reported, high expression of PD-L1, being statistically associated with more aggressive histological types, higher Masaoka-Koga stages and even worse prognosis (51–54), was observed in 23%-92% thymoma patients (52, 55–61). In addition, tumor-infiltrating lymphocytes, which are required for adequate activation of immune system, were diffusely and abundantly distributed in thymoma cases (60, 62). Taken together, high PD-L1 expression on tumor cells and abundant tumor-infiltrating lymphocytes in the microenvironment, provide a strong rational for implementing PD-1/PD-L1 therapy for thymomas to overcome the poor outcome results. Several trials have tentatively explored the efficacy and safety of immunotherapy in relapsed or advanced thymomas.

Cho et al. (63) performed a prospective phase II study to evaluate the role of pembrolizumab in thymoma patients who are refractory to initial standard platinum-based chemotherapy. A total of 7 thymoma patients were enrolled in the study and 2 of them (28.6%) achieved a partial response. Rajan et al. conducted a phase I trial with anti- PD-L1 antibody (Avelumab) in 7 advanced thymomas. Similar to the prior research, nearly 30% of the patients had an objective response (64). Due to the preliminarily promising results, a series of trials are on going to explore the role of immune therapy in relapsed or advanced thymomas (NCT03076554, NCT03134118, NCT03295227, NCT03463460, NCT02364076). In addition to efficacy, treatment toxicities are also important considerations. In the study of Cho et al., 5 (71.4%) of the 7 patients developed grade ≥ 3 immune-related adverse events, including 4 hepatitis and 3 myocarditis events. And in the research of Rajan et al., all of these patients that had tumor shrinkage developed immune related adverse events. Meanwhile, another article revealed that the administration of anti-PD1 immune check point inhibitor resulted in a storm of immune related adverse events (including myositis, myocarditis and myasthenia gravis and death) after administration of the first treatment cycle (65). Taken these limited data together, it looks that thymoma patients were at higher risk of developing immune-related side effects, so special caution is required for the usage of these agents in thymomas.

The exact role of induction immunotherapy in thymoma must be answered by clinical trials. To date, only two ongoing studies registered in the Chinese Clinical Trial Registry (ChiCTR2000036033) and clinicaltrials.gov (NCT03858582) are currently underway. All patients in the above two clinical trials will receive induction immunotherapy combined with chemotherapy. We hope these clinical trials will provide a conclusive answer to this question.

## Addition of Targeted Drugs to Preoperative Treatment

Similar to immunotherapy, evidence supporting targeted treatment in the neoadjuvant setting for thymoma is limited. We searched the database of Chinese Clinical Trial Registry and ClinicalTrials.gov and only one clinical trial was found. This ongoing phase II study (NCT01025089) is exploring the effects of the combination of cetuximab with traditional PAC scheme as neoadjuvant therapy for locally advanced thymoma. Patients will initially receive cetuximab weekly for up to 4 weeks to assess tumor response to cetuximab alone. Then, they will continue to receive weekly cetuximab along with concurrent CAP for 4 cycles before surgical treatment. The primary endpoint of the study is the frequency of a complete pathological response. The secondary endpoints include toxicity, treatment response, and R0 resection.

With the advances in the understanding of molecular biology, unique genetic aberrations associated with thymoma have been identified. All these specific biological markers involving KIT, EGFR, IGF-1R, and VEGF pathways, will facilitate the use of new targeted drugs in the future (66). Targeted drugs may be an option in clinical practice for heavily pretreated thymoma patients. Like immune therapy, targeted drugs alone cannot exert a sufficient impact on thymoma shrinkage in the setting of induction treatment. However, different from immunotherapy, the side effects of targeted drugs in thymoma may be well tolerated, based on current evidence and some agents have shown promising effects (67). Thus, the benefits of incorporating these targeted drugs into preoperative chemotherapy deserve further study.

## Discussion

This narrative review is presenting updated knowledge regarding induction strategy in initially unresectable thymomas. Based on current evidence, induction chemotherapy is still the mainstay in the induction setting.

The actual role of various chemo regimens needs to be answered in randomized clinical trial. However, under the circumstance of lacking enough patients, systematic review using the principle of evidence-based medicine may be an alternative way. Berghmans et al. evaluated the effectiveness of the different systemic therapies in the systemic treatments for thymoma. It revealed that cisplatin-anthracycline (PAC or ADOC) combinations were the most popular and active regimens (68). Similarly, the Italian Collaborative Group for Thymic Malignancies also recommends PAC scheme given the high rate of tumor shrinkage (69). Though, some differences may exist between induction and systemic chemotherapy, the results of the above systematic review, can also shed light onto the the choice of induction chemotherapy. Towards data shown in **Table 1**, the common type of induction chemo was platinum-anthracycline-based followed by platinum-etoposide-based schedules. Similar response rate, approximately 62%-100%, could be achieved by the two regimens. As for another chemo regimen (TP), the response rate was only 55.6%. Consequently, platinum-anthracycline-based or platinum-etoposide-based schedules should be proposed as front-line therapy.

In addition to efficacy, clinicians should also pay close attention to potential side effects, particularly cardiac toxicity. The risk of cardiotoxicity in patients with thymoma is very high due to a number of factors. Firstly, the tumor itself can invade the adjacent heart and great vessels. Secondly, the surgical procedure and radiotherapy can also exert a harmful impact on the heart (70, 71). Thirdly, cardiotoxicity can be caused by anthracycline treatment (72). Thus, for patients with old age, heart disease, and concomitant radiotherapy, great caution should be given to potential cardiotoxicity. Chemotherapeutic regimens without anthracycline, such as EP-based schemes, should be considered preferentially.

Although effective, chemotherapy alone sometimes is not powerful enough to make tumor remission. In these cases, additional radiotherapy, or chemoradiotherapy can present as salvage treatment modalities. Collectively, we propose the following strategies deserve to be tested in future studies. For patients with highly invasive features, such as great blood vessel invasion or B3 subtype, concurrent chemoradiotherapy may be a better induction strategy compared with chemotherapy alone (not in bulky tumors where radiation fields include a large proportion of the lung). For patients without such features, induction chemotherapy is preferred, and once tumors being refractory to chemotherapy, subsequent induction radiation should be considered.

Immunotherapy alone or in combination with chemotherapy is currently a revolution in the neoadjuvant treatment of non-small cell lung cancer (73–75). As for thymoma patients, will immunotherapy exert similar encouraging effects? From the available data, therapeutic prospects may be not very optimistic, because of limited activity of tumor control and high incidence of sever immune-induced toxicity. In our center, induction immunotherapy is only considered in thymomas, especially B3 subtype, refractory to induction chemotherapy or additional radiotherapy. So, the role of immunotherapy in induction treatment still requires confirmation.

Next generation sequencing and other advances in molecular biology have opened a new era for molecularly targeted therapies. From current data, targeted therapies in thymoma exhibited limited value and are only recommended to heavily pretreated advanced thymoma patients (67). However, different from immune or chemo therapy, the treatment-related toxicity of such agents is well tolerable in almost all reported cases. Thus, the benefits of incorporating these targeted drugs into preoperative chemotherapy deserve further study.

In general, given the rarity of the tumor, high-level clinical trials are difficult to perform. Thereby, in the future, collaborative efforts involving different organizations that specially focus on thymomas, such as the International Thymic Malignancy Interest Group (ITMIG) and Chinese Alliance for Research in Thymomas (ChART), should be promoted to encourage multicenter cooperation in clinical studies. Only in this way can we obtain enough sample sizes which will allow definitive conclusions about optimal treatment modalities.

## Author Contributions

Conception and design: SD and JD. Literature review and analysis: SD, JD, YZ, and ZL. Drafting of the manuscript: YZ, ZL, and YC. Supervision: LT and ZZ. All authors contributed to the article and approved the submitted version.

## Funding

This work was supported by the National Nature Science Foundation of China (82073479) and Scientific Research Project of Shanghai Science and Technology Commission (20ZR1410600).

## Conflict of Interest

The authors declare that the research was conducted in the absence of any commercial or financial relationships that could be construed as a potential conflict of interest.
